# Perceived burden and coping strategies among parents of children with autism spectrum disorder attending in tertiary hospital, Kathmandu

**DOI:** 10.1371/journal.pmen.0000254

**Published:** 2025-04-24

**Authors:** Isha Bhusal, Amar Regmi, Shobha Laxmi Bajracharya

**Affiliations:** 1 Lumbini Provincial Hospital, Rupandhehi, Nepal; 2 Province Health Logistic Management Centre, Rupandhehi, Nepal; 3 Patan Academy of Health Sciences (PAHS), Lalitpur, Nepal; Bowen University, NIGERIA

## Abstract

The parents of children with autism spectrum disorder face a mountain of practical problems. Helpful coping resources enable to restore equilibrium, otherwise, parents can perceive burden. The aim of the study was to find out the perceived burden and coping strategies among parents of children with autism spectrum disorder. This cross-sectional analytical study was conducted at Kanti Children’s Hospital, Kathmandu, Nepal among 78 parents of children with autism spectrum disorder selected by purposive sampling and interviewed using structured questionnaire. Zarit Burden Interview-22 tool was used to assess perceived burden and BRIEF COPE inventory tool was used to assess coping strategies among parents of children with autism spectrum disorder. Data analysis was done using Statistical Package for Social Sciences version 16. Frequency, mean, range and standard deviation were used to represent socio-demographic variables, clinical variables, perceived burden level and coping strategies. Chi-square test was used to find out association between socio-demographic variables and perceived burden level. Spearman rank correlation was used to find out relationship between perceived burden and coping. Mild burden was perceived by 42.3%, moderate perceived by 39.7%, severe perceived by 10.3% and no burden were perceived by 7.7%. The mean score (26.85) was higher at emotional focused coping strategies. No association was found between demographic factors and level of burden. Emotional focused coping (r = 0.288, p = 0.01) was positively correlated with overall burden.Majority of parents had mild burden while more than one third had moderate burden. The most popular coping mechanism, emotional focused, had a positive correlation with overall burden.

## Introduction

Autism Spectrum Disorders (ASDs) are neurodevelopmental conditions that involve severe impairment in social interactions, communication skills, and presence of restricted stereotypical behaviors. One in 160 children have ASD globally [1]. Several studies demonstrated high level of burden in caregivers of autistic children [2–4].

People with autism can have different needs and abilities and can evolve over time. Some autistic people can live safe sufficiently in their own, whereas autistic people with severe disability require endless care and support throughout their lifetime [1]. Parents’ perceived burden increases as they struggle to find suitable services for their children. As ASD impacts the whole family, extra services are required to meet the family needs but service providers often lack specialized training to meet the special needs of the ASD children and their parents [5]. Studies have shown high level of stress in families of children with ASD, with mother being the primary target and emotional strain of fathers regarding financial and occupational difficulties [6]. From the early age, child with ASD puts a great challenge in the parenting skills. If parents are not able to utilize the helpful coping resources and parents’ perceptions of the demands of their parental role exceed without being able to restore an equilibrium through the usual methods and strategies, they can experience a complex array of stressors [7].

A study conducted in Australia explored the coping strategies of parents raising a child with ASD which found different coping strategies among mother and father. Mothers reported greater use of emotion-focused strategies like denial, avoidance, or venting while father used problem-focused strategies [8]. A study in India among 90 caregivers pointed out that the majority (64.4%) of caregivers perceived high burden (Median = 71.87 with interquartile range of 63.28-77.08) [9]. Another study in India among 63 parents showed gender of child and parents’ age, gender, level of education & Income affected parental perceived burden. The study also revealed a negative impact on multiple areas of life [10]. In Nepal, among 61 caregivers at Autism Care Nepal Society, almost half of the caregivers of children with ASD (42.6%) experience moderate to severe burden [11]. Parents’ well-being could positively influence the care giving process and decrease the burden of care [12].

Within this context, this study aims to find out the perceived burden and coping strategies among parents of children with autism spectrum disorder and examine the association between demographic variables of parents and perceived burden. The findings from this study may provide baseline data related to perceived burden and coping strategies. This may enable nurses to emphasize their role in counselling of the parents, along with the care of children with ASD. Furthermore, the study might help nurses to become mindful on the various coping strategies such as problem focused, emotion focused and avoidant coping.

## Materials and methods

### Study design

A Cross-sectional analytical research design was adopted for the study to assess the burden perceived by parents of ASD child and their coping strategies.

### Study site

The site of the study was the Child and Adolescent Mental Health (CAMH) unit at Kanti Children’s Hospital (KCH), Kathmandu, Nepal. Child and Adolescent Mental Health (CAMH) unit at Kanti Children’s Hospital (KCH) has been providing CAMH services since 2015. This is the only full-time child and adolescent psychiatry outpatient clinic (CAP OPD) in Nepal.

### Study duration

The study was conducted from August 12, 2021 to April 16, 2023. Participant recruitment took place from July 24, 2024 to September 3, 2022.

### Sampling procedure and sample size

A purposive sampling technique was used to select study participants. The sample size of 78 was determined using Cochran’s formula [13] to ensure adequate statistical power.

The sample size was determined by using the following Cochran formula: -


$$n=\;\frac{\frac{z^{2}pq}{{\text{e}}^{2}}}{1+\left[\frac{z^{2}pq}{e^{2}N}\right]}$$

Where,

n = required sample size

z = standard value of z sore at 5% level of significance = 1.96

p = 32.23% burden [11] =32.23/100= 0.32

q = (1-p) =1-0.32= 0.68

e = margin of error at 5%=0.05

Based on the record of child and adolescent psychiatry outpatient clinic of Kanti Children’s Hospital (N) =90

Using the formula for finite population,


$$n-\;\frac{\frac{z^{2}pq}{{\text{e}}^{2}}}{1+\left[\frac{z^{2}pq}{e^{2}N}\right]}$$

$$n=\frac{\frac{{\left(1.96\right)}^{2}*0.32*0.68}{{\left(0.05\right)}^{2}}}{1+\frac{\left[{\left(1.96\right)}^{2}0.32*0.68\right]}{{\left(0.05\right)}^{2}*90}}$$

$$n=\;\frac{\frac{0.835}{0.0025}}{1+\frac{0.84}{0.225}}$$

$$n=\frac{334.37}{4.7}\;=71+10\%=78$$

Considering the non-response rate of 10%, the final sample size was estimated.

### Study participants

The study population consisted of all the parents either father or mother who were caring for ASD child attending in child and adolescent psychiatry outpatient clinic (CAP OPD) during the study period.

The Inclusion criteria for the study were parents who were willing to participate and gave consent, parents who were 18 years or older, and either father or mother who lived with the child in the same house and had been continuously taking care of the autistic child/adolescent for at least the past three months.

### Study variables

The study variables has include dependent and Independent Variables. Independent variables included Socio-demographic variables of parents & child and disease condition.

#### Socio-demographic variables.

Parents’ age, gender, family type, educational level, termination of job after diagnosis, age of child, gender of child, education of child & training received by parents.

#### Clinical variables of children.

Duration of illness & co-morbidity

#### Dependent/ outcome variable.

Level of Perceived Burden- (No burden: Little or no burden/Burden: Mild,Moderate & Severe); Coping Strategies- Problem Focused,Emotion Focused, Avoidant Coping strategies.

### Data collection and analysis

Data was collected through face to face interview using structured interview questionnaire. Interview was taken during the waiting time before and after the parent takes child to be seen by doctor and clinical psychologist. Data was collected by researcher herself after the written consent obtained from the respondents. The purpose of the study, risk and benefits were well explained to the respondents participating in the study. Allocating about 20–25 minutes for each respondent, approximately 2–3 parents were interviewed from 9 am to 3 pm from Sunday to Friday for six weeks except public holidays. Data was collected at the separate corner of the waiting hall in OPD. Eighty four respondents were approached. Six refused to participate in the study. After data collection informal health teaching and counseling regarding care of ASD child was given to parents.

Data collection instrument of this study was the structured interview schedule in Nepali language with following parts; Part I: Socio demographic variables of parents & children and disease condition that consisted 12 items. In this part, questions about parents’ age, gender, family type, educational level, termination of job after diagnosis, age of child, gender of child, education of child, duration of illness, co-morbidity, training received by parents were included; Part II: Zarit Burden Interview-22(ZBI-22) was used in this study to assess perceived burden among parents of children with Autism spectrum disorder (ASD). Zarit Burden Interview-22 (ZBI-22) is composed of 22 items questionnaire under five domains, i.e., burden in relationship (6 items), emotional well-being (7 items), social and family life (4 items), finances (1 item) and loss of control over one’s life (4 items). It consists of five-item response that reflect how a person feels when they are taking care of another person. The total score range from 0 to 88. A total score was computed by summing the 22 items and high scores indicate a higher level of burden. Level of burden were measured in 4 levels: Little or no burden (0–20), Mild burden (21–40), Moderate burden (41–60), Severe burden (61–88). [14]; and Part III: BRIEF COPE inventory was used in this study to assess coping strategies among parents of children with Autism spectrum disorder (ASD) [15]. The BRIEF-COPE is a 28 item questionnaire developed by Carver (1977) which is an abbreviated version of the COPE inventory. The Brief COPE consists of 14 scales of two items each with 28 items. It consists of 14 scales under three major domains. The three domains are problem-focused, emotion-focused and avoidant coping strategies. The ZBI-22 & BRIEF COPE has been widely used and well validated in numerous previous studies, with a Cronbach’s α of ZBI-22 is 0.92 in the original scale [16]. The Cronbach’s alpha for the original subscales of BRIEF COPE ranged from 0.50 (venting) to 0.90 (substance use) [15]. The Cronbach’s alpha in this study for Zarit Burden Interview-22 (ZBI-22) is 0.82 and BRIEF COPE inventory is 0.71 which indicated acceptable internal consistency of both tools. Statistical Package for Social Sciences (SPSS) version 16 was used for data processing and analysis.

Frequency and percentage were used to represent socio-demographic variables, clinical variables and perceived burden level whereas mean, range and standard deviation were used to represent coping strategies used by parents. Mean and standard deviation was also used to represent age and duration. Assumption test for chi-square (goodness of fit) was assessed [17]. It was achieved. So, the association between socio-demographic variables and level of perceived burden were analyzed using Chi-square test. Statistical significance was considered at p < 0.05 and p-value of the test has been included in the respective tables. Assumption test for correlation (linearity) was done [18]. Since, assumption test of correlation did not meet, Spearman Rank Correlation was used to find out the relationship between perceived burden and coping.

### Ethical Consideration

Ethical approval was taken from the Institutional Review Committee of Patan Academy of Health Sciences (Ref: PNM2206211645) and Institutional Review Board (IRB) of Kanti Children’s Hospital (Ref: 770/78–79) for the study. The written consent was obtained from each respondent (parent) before the interview study for which the generic PAHS format in Nepali was used. Respondents were allowed to withdraw from the study at any time without giving any reason during the study period.

## Results

The mean age of the respondents was 31.76 ± 5.28 years. Sixty (76.92%) respondents in the study were mothers, 46(58.97%) were from nuclear family, 29(37.18%) had secondary level education, 62(79.49%) had not received any training about rearing a child of ASD and 68 (87.18%) of them did not have to terminate the job after diagnosis of their child with ASD. (Table 1) Similarly, 62(79.49%) of children belonged to age group 2–5 years, 66 (84.62%) were male child and 38 (48.72%) of them were attending normal school. (Table 2).

**Table 1:** Socio-demographic characteristics of respondents.

Socio-demographic Variables of Parents	Frequency (n)	Percent (%)
**Age (in completed years**)		
Mean ± SD	31.76 ± 5.28	
Minimum to Maximum	20–47	
**Gender**		
Mother	60	76.92
Father	18	23.08
**Family type**		
Nuclear	46	58.97
Joint	30	38.46
Extended	2	2.57
**Education**		
No education	2	2.56
Can read & write	2	2.56
Primary	7	8.98
Secondary	29	37.18
Bachelor	25	32.05
Master’s & above	13	16.67
**Termination of Job**		
Yes	10	12.82
No	68	87.18
**Training received by parents about rearing a child of ASD**		
Yes	16	20.51
No	62	79.49

**Table 2:** Socio-demographic characteristics of children with autism spectrum disorder.

Socio-demographic Variables of Children	Frequency (n)	Percent (%)
**Age (Completed years)**		
2-5	62	79.49
6-11	15	19.23
12 and above	1	1.28
Mean ±SD	4.45 ± 1.89	
Minimum to Maximum	2–12	
**Gender**		
Male	66	84.62
Female	12	15.38
**Education**		
Not attending	31	39.74
Attending normal school	38	48.72
Attending special education	9	11.54

Among 78 children with autism, majority (76.9%) of them were diagnosed for less than or equal to 12 months. Highest percentage of children (94.9%) did not have any co-morbidities. (Table 3).

**Table 3:** Clinical variables of children.

Clinical Variables	Frequency (n)	Percent (%)
**Duration of illness after diagnosis**		
≤ 12 months	60	76.9
> 12 months	18	23.1
Median with IQR	11.50 (6)	
Min to Max (months)	3–120	
**Co-morbidities of Child**		
Not present	74	94.9
ADHD	1	1.3
Intellectual Disability Disorder (IDD)	1	1.3
Myopia	1	1.3
Seizure disorder	1	1.3

Perceived burden of respondents shows that 26.9% of respondents had sometimes felt about asking for more help than child required. Similarly 32.1% of them had quite frequently felt that they don’t have enough time for themselves because of caring. Likewise, 32.1% of respondents had sometimes felt stressed between caring and trying to meet other responsibilities. One fourth of the respondents (25.6%) had never felt embarrassed over their child’s behavior whereas another one fourth (25.6%) had quite frequently felt embarrassed over their child’s behavior.

More than one third of respondents (35.9%) had never felt that they don’t have enough money to care for child, in addition to the rest of their expenses. More than half of the respondents (59%) had never felt they will be unable to take care of their child much longer. Similarly, more than one third (34.6%) had never felt lost control of life since child’s illness. Two third (66.7%) had never wish they could just leave the care of child to someone else. More than one fourth of them (29.5%) had quite frequently felt uncertain about what to do about child.

Similarly, more than half of the respondents (59%) had nearly always felt they should be doing more for their child. Whereas more than two third of respondents (67.9%) had felt nearly always a better job in caring for child. Similarly, more than half of the caregivers (51.3%) had sometimes felt burdened in caring for their child. (Table 4).

**Table 4:** Perceived burden of respondents.

Items	0	1	2	3	4
	n(%)	n(%)	n(%)	n(%)	n(%)
Asks for more help than required	4(5.1)	19(24.4)	21(26.9)	20(25.6)	14(17.9)
Don’t have enough time for yourself	12(15.4)	13(16.7)	17(21.8)	25(32.1)	11(14.1)
Feel stressed between caring and trying to meet other responsibilities	7(9.0)	19(24.4)	25(32.1)	19(24.4)	8(10.3)
Feel embarrassed about child’s behaviour	20(25.6)	16(20.5)	12(15.4)	20(25.6)	10(12.8)
Feel angry around your child	24(30.8)	12(15.4)	24(30.8)	14(17.9)	4(5.1)
Affects your relationship with family members or friends in a negative way	31(39.7)	19(24.4)	12(15.4)	9(11.5)	7(9.0)
Afraid of the future of child	3(3.8)	6 (7.7)	13(16.7)	11(14.1)	45(57.7)
Feel child is dependent upon you	6(7.7)	16(20.5)	20(25.6)	18(23.1)	18(23.1)
Feel tense when around your child	20(25.6)	14(17.9)	19(24.4)	14(17.9)	11(14.1)
Feel your health has suffered	34(43.6)	19(24.4)	19(24.4)	4(5.1)	2(2.6)
Feel you don’t have much privacy	9 (11.5)	11(14.1)	18(23.1)	29(37.2)	11(14.1)
Feel that social life has suffered	12(15.4)	12(15.4)	25(32.1)	23(29.5)	6(7.7)
Feel uncomfortable about having friends over because of your child	28(35.9)	10(12.8)	20(25.6)	9 (11.5)	11(14.1)
Feel that child expect you to take care of him or her	25(32.1)	29(37.2)	18(23.1)	4(5.1)	2(2.6)
Feel you don’t have enough money to care for your child	28(35.9)	12(15.4)	12(15.4)	13	13
Feel that you will be unable to take care of your child much longer	46(59.0)	16(20.5)	10(12.8)	5(6.4)	1(1.3)
Feel you have lost control of your life since your child’s illness	27(34.6)	26(33.3)	15(19.2)	10(12.8)	–
Wish you could just leave the care of your child to someone else	52(66.7)	14(17.9)	10(12.8)	2(2.6)	–
Feel uncertain about your child	8 (10.3)	14(17.9)	16(20.5)	23(29.5)	17(21.8)
Feel you should be doing more	1 (1.3)	3 (3.8)	12(15.4)	16(20.5)	46(59.0)
Feel you could do a better job in caring for your child	2 (2.6)	3 (3.8)	9 (11.5)	11(14.1)	53(67.9)
Overall burdened you feel in caring	7 (9.0)	22(28.2)	40(51.3)	8(10.3)	1(1.3)

Nearly half, i.e., 33 (42.31%) of the respondents had mild level of burden while more than one third, i.e., 31(39.74%) had moderate level of burden followed by severe burden 8(10.26%). Only 6(7.69%) respondents felt little or no burden. (Table 5) The mean score (26.85) was higher at emotional focused domain of coping strategies for respondents followed by problem focused (23.53) and avoidant coping (11.42). Specifically, positive reframing was mostly used (6.31), (Table 6).

**Table 5:** Levels of perceived burden of respondent.

Level of burden	Frequency (n)	Percent (%)
Little or no burden (0–20)	6	7.69
Mild (21–40)	33	42.31
Moderate (41–60)	31	39.74
Severe burden (61 and above)	8	10.26
**Mean score ± SD**	40.82 ± 15.19	
**Minimum to maximum**	8–74	

**Table 6:** Coping strategies score of respondents.

Coping strategies	Range	Mean	SD
**Problem focused**	**20**	**23.53**	**4.72**
Active coping	6	5.58	1.67
Use of informational support	6	6.13	1.52
Positive reframing	6	6.31	1.49
Planning	6	5.51	1.83
**Emotional focused**	**23**	**26.85**	**4.47**
Emotional Support	6	5.33	1.47
Venting	6	4.42	1.56
Humor	3	2	0.28
Acceptance	6	5.99	1.99
Self-blame	6	3.74	1.41
Religion	6	5.36	1.81
**Avoidant coping**	**11**	**11.42**	**3.03**
Self-distraction	6	3.96	1.81
Substance Use	6	2.29	1.01
Denial	6	2.92	1.36
Behavioral disengagement	6	2.24	0.96

The socio-demographic variables were checked for association with level of burden.From the analysis, there was no association found with any demographic factors and level of burden. (Table 7).)

**Table 7:** Association between level of perceived burden and socio-demographic variables.

Socio-demographic variables	Level of Burden		Chi-square	p-value
	Little to mild burden (0–40)	Moderate to severe burden(41–88)		
**Gender of parents**				
Father	12 (66.67%)	6 (33.3%)	2.6	0.1
Mother	27 (45.00%)	33 (55.00%)		
**Education of parents**				
Less than secondary or secondary	17 (42.50%)	23 (57.50%)	1.85	0.17
Bachelor and above	22 (57.89%)	16 (42.11%)		
**Gender of child**				
Male	31 (47.00%)	35 (53.00%)	1.58	0.21
Female	8 (66.67%)	4 (33.33%)		

Relationship between overall perceived burden and coping strategies were analyzed. We found that the Emotional focused coping (r = 0.288) had weak positive relationship with overall burden. (Table 8).)

**Table 8:** Relationship between overall perceived burden level and coping strategies.

Spearman’s rank correlation					
	1. Overall coping	2. Problem focused	3. Emotional focused	4. Avoidant	5. Overall burden
1.Overall coping	–				
1.Problem focused	0.823^**^(0.00)	–			
2.Emotional focused	0.833^**^(0.00)	0.537^**^(0.00)	–		
3.Avoidant	0.309^**^(0.00)	0.073(0.52)	0.054(0.64)	–	
4.Overall burden	0.126(0.27)	0.003(0.98)	0.288^*^(0.01)	-0.054(0.64)	–

*Note: Number in parenthesis represent p-value*

* Correlation is significant at the < 0.05 level (2-tailed).

** Correlation is significant at the < 0.01 level (2-tailed).

## Discussion

In this study, mean burden score was 40.82 ± 15.19. The categorization of burden revealed 42.3% of respondents had mild, 39.7% had moderate and 10.3% had severe burden. Only 7.7% of them felt no or little burden. Similar finding is reported by a study done among 84 Pakistani parents of autistic children which showed higher caregiving burden with mean burden score 41.75 ± 10.62 and only 3.6% of them reported no burden [19]. Contradictory finding is shown by a study done in Indonesia among 24 mothers of ASD children that revealed that only 8% of them had high objective burden and no participant with very high level objective burden [20]. This might be due to difference in the cultural and supportive context of the two countries. Due to unfavorable economic conditions, there is not enough comprehensive support from government for these parents that may affect the severity of the care burden imposed on these parents.

Findings of this study showed emotional focused coping and problem focused coping was mostly used coping strategies by parents. Specifically, from the problem focused domain, (positive reframing, use of informational support, active coping) and emotional focused domain (acceptance and religion) was mostly used coping strategies by parents. Similar findings were found in a study done in New Zealand among 178 parents reported most likely used coping from the cognitive reframing (acceptance, positive reframing, religion) and engagement (active coping planning) dimensions [21]. This finding is also in line with the study done in India among 30 mothers which showed that the major coping styles adopted by the mothers were active coping (38.3%), positive reframing (38.3%), planning (38.3%), religious coping (38.3%) and acceptance (36.65%) [22]. Contradictory findings with this study was observed in study done in Iran among 43 parents shows that avoidant coping style (37%) was the widely used strategy [23]. This contrast may be due to the difference in perception of stress & burden especially in the setting of a different country.

None of the selected socio-demographic characteristics of parents & child were found to have any association with perceived parental burden. The findings of this study showed that there was no statistical association of perceived burden and gender of parents (p = 0.10). However, study found that parents who were female felt higher burden (55%) than male (33.3%). It might be because in Nepali culture, women are regarded as primary caregivers and are predominantly involved in caregiving and have high care load while putting their health and well-being worse off that ultimately leads to more burden & stress. Similar findings were found in study done in Italy among 359 parents revealed no significant association (p = 0.11) between parental objective burden and gender of parents [3]. The findings of this study contradicted with a cross-sectional study done in Iraq among 152 participants which showed that there is a significant relation (p = 0.004) between the caregiver genders with psychosocial burden of caregiver [24].

The findings of this study showed that there was no statistical association of perceived burden and level of education of parents (p = 0.17). However, the study found that parents who completed bachelor level education felt lower burden (57.9%) than those whose educational level was secondary level & below (42.5%). It is a general consideration that increased education and awareness about a disease condition may make it more tolerable for patients and their families. This finding is in line with the study done in Japan among 26 mothers of children with ASD which showed there was no significant association (p = 0.093) of caregiver burden and education [20]. However, on the contrary to this study, a study done in India showed a statistically significant association (p = 0.018) between caregiver burden and educational level of the parents [25].

The findings of this study showed that there was no statistical association of parental perceived burden and child’s gender. However, findings of this study showed that parents of male child felt more burden than parents of female child. This might be because of more dependency of male child in activities of daily living than female child. This is supported by the study done in Nepal among 61 parents in which it has been found that gender of child doesn’t affect parental perceived burden (p = 0.577) [11]. However, this finding contradicts with the findings of the study done in Turkey among 131 parents which showed that parents of female patients had significantly higher burden (p = 0.033) [26].

The findings of this study showed that overall perceived burden is positively correlated with emotional focused coping. Similar study in Taiwan among 60 mothers shows that positive correlation was found between the overall burden and emotion-focused coping [27]. A cross-sectional study conducted with 131 parents of children with ASD in Turkey reported that there was a significant statistical correlation between caregiver burden and coping strategies [26]. The reason for this similarity could be the use of the same tools to assess coping strategies in parents of autistic children. While contrast to this study, a study done in Iran among 134 mothers showed that there was inverse correlation found to exist between caring burden with coping strategies [28].

## Strengths and Limitations

This study was a cross-sectional analytical design which provided valuable insights into associations between dependent and independent variable. The use of validated tools and techniques correctly has added the strength and made this study more valid.

The tool of my study restricted the ability to explore potential burden, thus lacking to reflect detail provided by participants. The sample selection of the study was purposive which lacks randomization. Hence, this limits the generalizability of the findings. Another limitation is self-selection bias which also limits generalizability, parents who volunteered to participate in the study may have been less burdened than those who did not respond. Another limitation of this study is the small sample size which was constrained by the limited number of ASD cases available at Kanti Children’s Hospital.

## Conclusions

The findings of the study showed that majority of the caregivers had mild level of burden while more than one third had moderate level of burden. Similarly, Emotional focused and problem focused coping were the most commonly used coping strategies by parents. Specifically, the use of positive reframing was the most preferred coping strategy. Emotional focused coping was positively correlated with overall burden. The study findings might help health care providers such as nurses, doctors and public health personnel as well as family members to be aware about parental burden and target them early in the caregiving process before they suffer negative outcomes which has a broader scope in mental health. This study might have potential to help health personnel to plan for necessary parental training program to increase child’s adaptive behaviors which ultimately minimizes the burden among parents and maximize the coping ability in context of Nepal. Further research with qualitative method can be done to explore the burden experienced by parents of child with ASD.

## Supporting information

S1 Table Test of normality. (PDF)
