# Supplementary material for: Perceived burden and coping strategies among parents of children with autism spectrum disorder attending in tertiary hospital, Kathmandu
Source: PLOS Ment Health. 2025 Apr 24;2(4):e0000254. doi: 10.1371/journal.pmen.0000254 (PMC12798197; doi:10.1371/journal.pmen.0000254)
Supplement: S1 Table — (PDF) [file pmen.0000254.s001.pdf]

S1 Table: Test of Normality

	Burden	Coping
**Skewness**	-0.207	-0.172
**Standard Error of Skewness**	0.347	0.272
**Z- Skewness**	-0.597	-0.632

Note: Z score for skewness = Skewness/Standard error

Data is normally distributed (Kim, 2013)

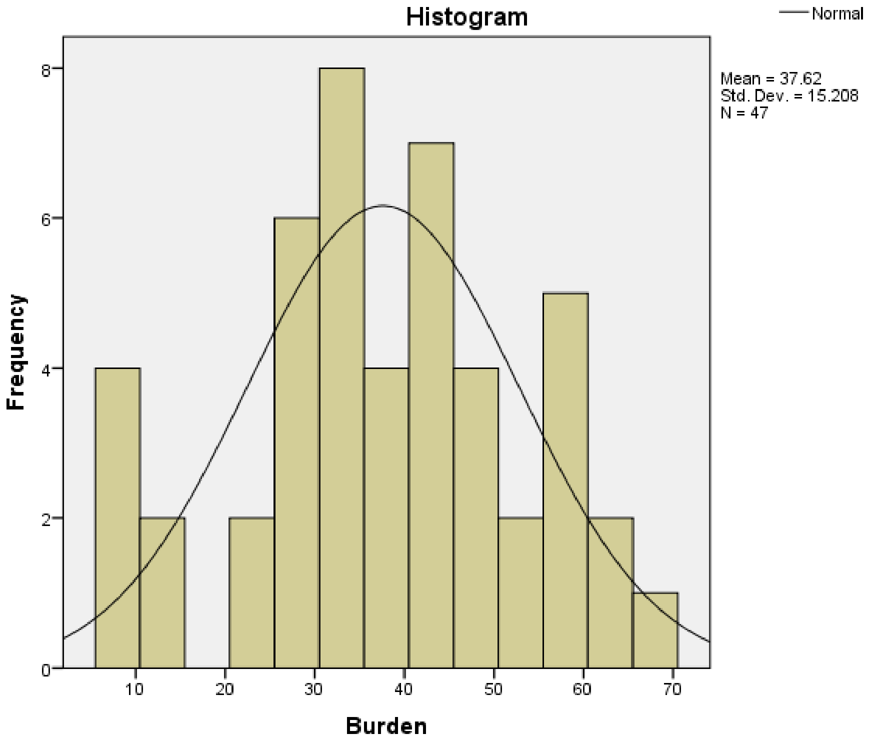

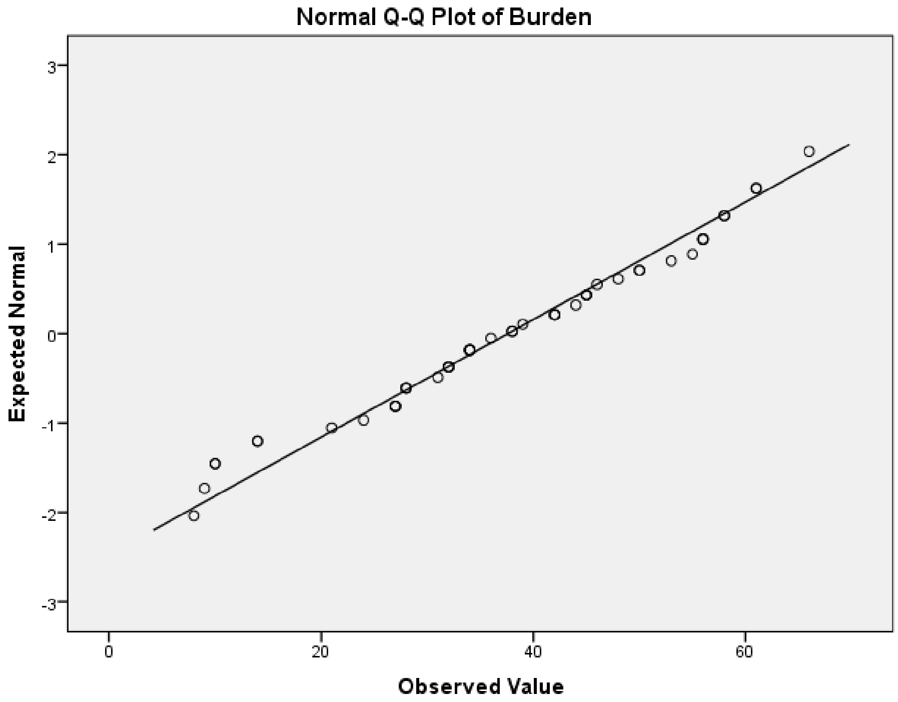

Normal Q-Q Plot of Burden
Expected Normal
Observed Value
3
2
1
0
-1
-2
-3
0
20
40
60

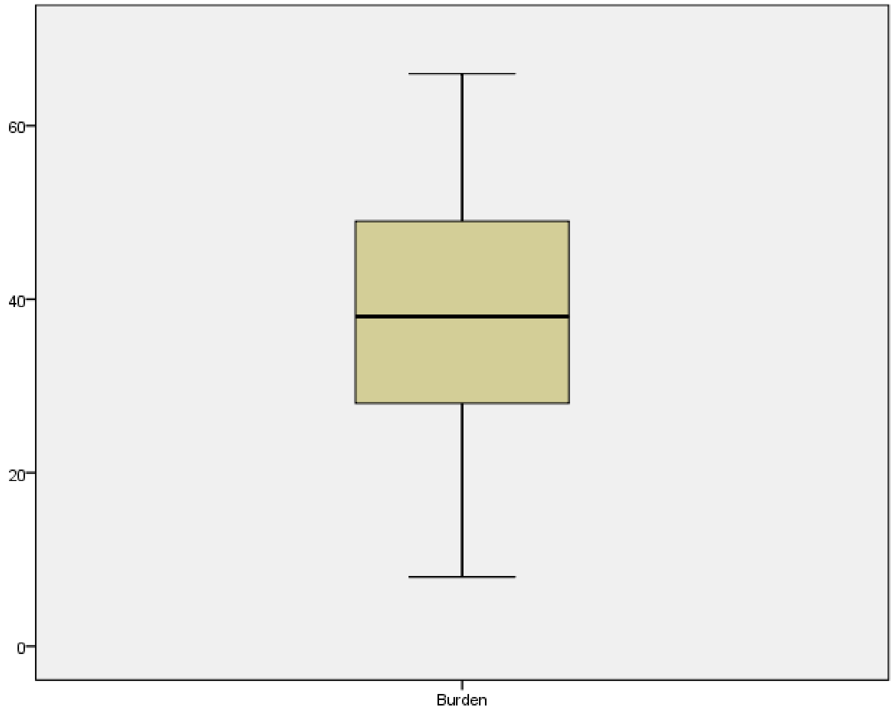

60
40
20
0
Burden

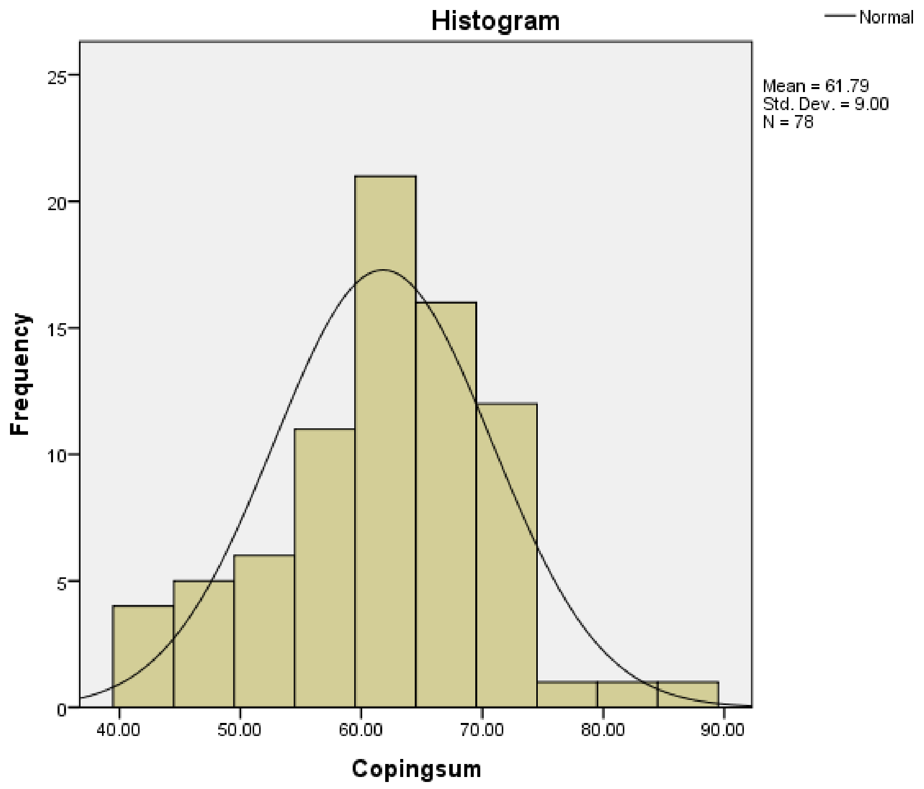
Histogram
Normal
Mean = 61.79
Std. Dev. = 9.00
N = 78
Frequency
25
20
15
10
5
0
40.00
50.00
60.00
70.00
80.00
90.00
Copingsum

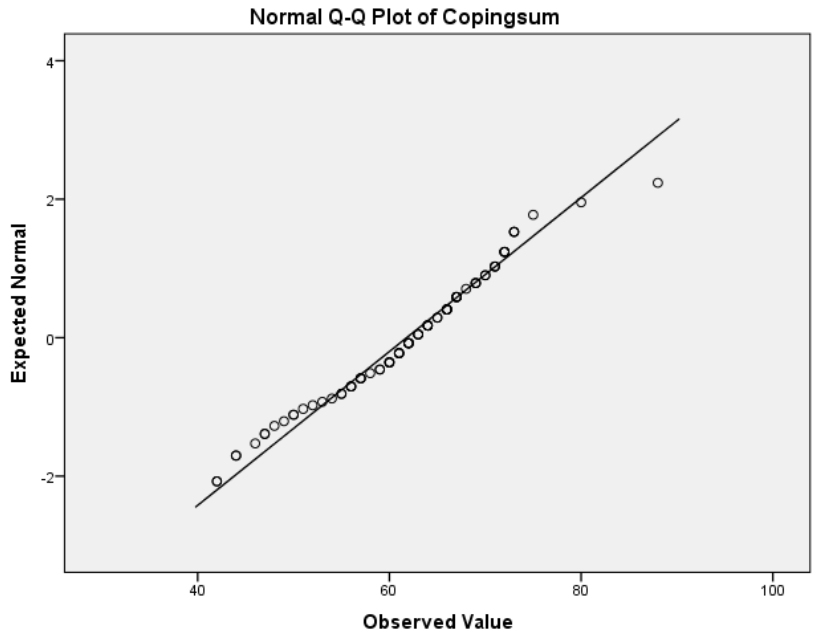
Normal Q-Q Plot of Copingsum
Expected Normal
4
2
0
-2
40
60
80
100
Observed Value
